# Antibiotic-resistant organisms establish reservoirs in new hospital built environments and are related to patient blood infection isolates

**DOI:** 10.1038/s43856-022-00124-5

**Published:** 2022-06-01

**Authors:** Kimberley V. Sukhum, Erin P. Newcomer, Candice Cass, Meghan A. Wallace, Caitlin Johnson, Jeremy Fine, Steven Sax, Margaret H. Barlet, Carey-Ann D. Burnham, Gautam Dantas, Jennie H. Kwon

**Affiliations:** 1 The Edison Family Center for Genome Sciences and Systems Biology, Washington University School of Medicine in St Louis, St Louis, MO USA; 2 Department of Pathology and Immunology, Washington University School of Medicine in St Louis, St Louis, MO USA; 3 Department of Biomedical Engineering, Washington University in St Louis, St Louis, MO USA; 4 Department of Medicine, Washington University School of Medicine in St Louis, St Louis, MO USA; 5 Department of Molecular Microbiology, Washington University School of Medicine in St Louis, St Louis, MO USA; 6 Department of Pediatrics, Washington University School of Medicine in St Louis, St Louis, MO USA

**Keywords:** Comparative genomics, Infectious-disease epidemiology, Antimicrobial resistance, Disease prevention, Genetic variation

## Abstract

**Background:**

Healthcare-associated infections due to antibiotic-resistant organisms pose an acute and rising threat to critically ill and immunocompromised patients. To evaluate reservoirs of antibiotic-resistant organisms as a source of transmission to patients, we interrogated isolates from environmental surfaces, patient feces, and patient blood infections from an established and a newly built intensive care unit.

**Methods:**

We used selective culture to recover 829 antibiotic-resistant organisms from 1594 environmental and 72 patient fecal samples, in addition to 81 isolates from blood cultures. We conducted antibiotic susceptibility testing and short- and long-read whole genome sequencing on recovered isolates.

**Results:**

Antibiotic-resistant organism burden is highest in sink drains compared to other surfaces. *Pseudomonas aeruginosa* is the most frequently cultured organism from surfaces in both intensive care units. From whole genome sequencing, different lineages of *P. aeruginosa* dominate in each unit; one *P. aeruginosa* lineage of ST1894 is found in multiple sink drains in the new intensive care unit and 3.7% of blood isolates analyzed, suggesting movement of this clone between the environment and patients.

**Conclusions:**

These results highlight antibiotic-resistant organism reservoirs in hospital built environments as an important target for infection prevention in hospitalized patients.

## Plain Language Summary

Patients in hospitals often have a suppressed immune system, putting them at increased risk of infection by bacteria that are resistant to antibiotics, some of which may come from sources in the hospital environment. We sampled multiple different surfaces in an established and a newly built intensive care unit and collected patient infection samples. We tested bacteria in these samples for their resistance to antibiotics and sequenced the genetic code of the bacteria to identify relationships between environmental and patient infections. We found the most antibiotic resistant organisms in hospital sink drains. Our sequencing data revealed strains of a certain kind of bacteria could form reservoirs and survive in sink drains and also cause patient infections. These results highlight the importance of removing these antibiotic resistant organism reservoirs to prevent infections.

## Abstract (web summary)

Sukhum, Newcomer et al. evaluate reservoirs of antibiotic-resistant organisms within the built environment and patient samples from an established and a newly-built intensive care unit. The authors demonstrate colonization of sink drains and other sites and show relatedness between environmental reservoirs and patient infections.

## Introduction

Healthcare-associated infections (HAIs) are a global challenge, posing a particularly acute threat in intensive care units (ICUs) where critically ill and immunocompromised patients are at elevated risk for infection during their stay^1,2^. Worldwide, HAIs are responsible for an estimated 2.5 million infections every year and are associated with increased morbidity, mortality, and healthcare costs^1,3–5^. The COVID-19 pandemic is associated with further expansion of hospitalized critically-ill individuals^6^. HAIs due to AROs in the ICU can be difficult to treat due to limited treatment options; available options are also associated with toxicity, are poorly tolerated by patients, and may exhibit negative interactions with other drugs^1,7,8^.

Many studies and initiatives have focused on trying to limit HAIs through surveillance, prevention, and intervention^1,9,10^. Recent studies have used culture-independent metagenomic sequencing of hospital surfaces to generate an important catalog of the diversity and composition of their resident microbial communities^11–15^. However, metagenomic characterizations are limited in their ability to track viable, antibiotic-resistant strains and remain ambiguous to whether the taxa discovered on surfaces are environmental- or patient-derived, and/or associated with infections in patients. To better understand relationships between viable antibiotic-resistant organisms (ARO) in the built environment and critically-ill patients, we must determine 1) what hospital surfaces are acting as ARO reservoirs, i.e., surfaces where an organism can be cultured from multiple time points; 2) what are the spatial and temporal dynamics of reservoir colonization; and 3) whether viable ARO strains colonizing the hospital built environment can also be detected from human clinical infections.

**Fig. 1:**
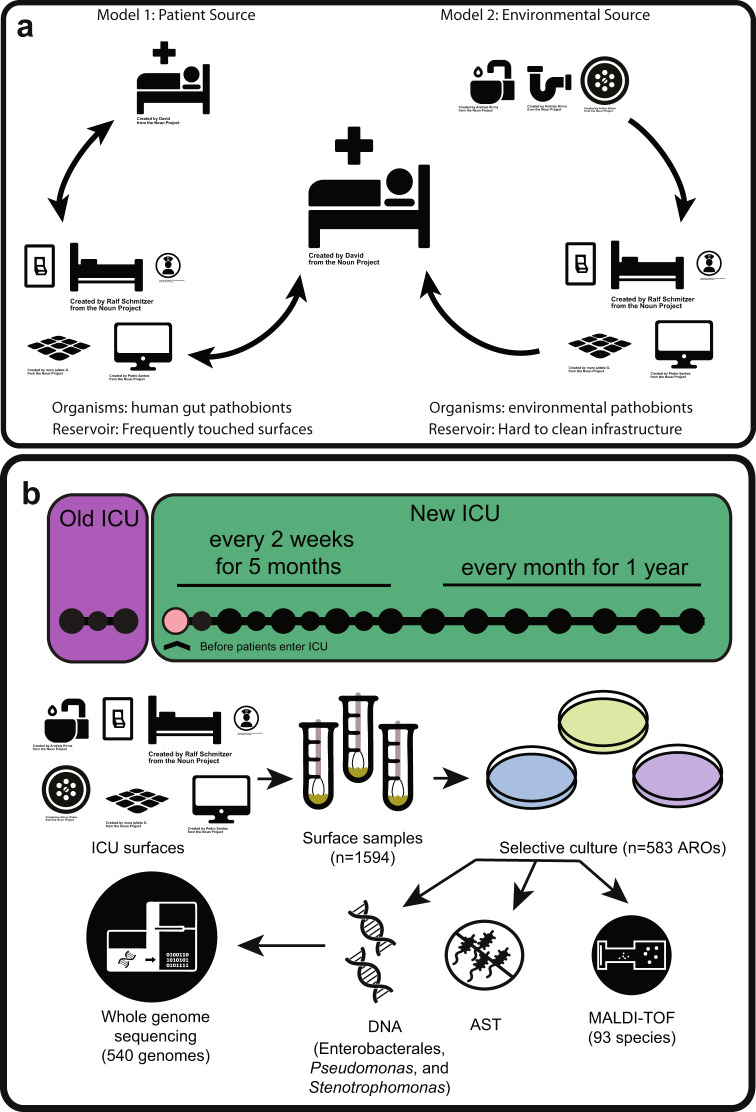
ARO reservoir colonization models and sample processing scheme. **a** Two models of reservoir colonization. Model 1 shows antibiotic-resistant organism (ARO) transmission from patients to hospital surfaces and then to other patients. Model 2 shows ARO transmission from environmental reservoirs to hospital surfaces to patients. **b** Sample collection time points and sample processing scheme from surface collections to WGS. In sample collection scheme, large circles represent months with small circles representing 2-week sampling within months. Purple indicates old intensive care unit (ICU) collections, green indicates new ICU collections, and pink indicates collections taken before patients enter the building in the new ICU. Icons labeled as such were acquired from nounproject.com, and other icons were used with permission from D’Souza, Potter et al.^82^. AST antibiotic susceptibility testing, MALDI-TOF matrix-assisted laser desorption/ionization-time of flight mass spectrometry.

There are multiple models proposed for ARO reservoir colonization and transmission in hospitals (Fig. 1a)^1,16–18^. A prominent model is that AROs are shed from colonized patients, frequently through fecal contamination, to surfaces, instruments, and shared equipment in patient rooms (Fig. 1a)^19,20^. High-touch hospital surfaces can act as intermediate ARO reservoirs, and transmission may occur from these reservoirs through patients, healthcare staff, and visitors^10,20–23^. Another model is that AROs are seeded from microbial communities which persistently colonize hospital built environments, particularly plumbing sources, where biofilms form and can act as a reservoir for potential pathogens (Fig. 1a)^24–27^. These models are not mutually exclusive. ARO reservoirs are likely dependent on a given facility’s history and modes of transmission likely interact within a hospital^28^. To better understand the colonization and transmission of AROs in the hospital built environment, we leveraged a unique opportunity to sample a newly-built stem cell transplant and oncology (SCT) ICU both before patient and staff occupancy and for one year after ICU establishment. This allowed us to identify and track persistent colonization of sink drains by AROs that began prior to patient and staff occupancy, a facet that has not been characterized in previous studies. As immunocompromised cancer patients demonstrate prolonged duration of ARO shedding and are at high risk of HAIs, the SCT ICU is a critical environment to study ARO surface colonization and transmission^29–32^. Additionally, we compared this new ICU environment (new ICU) with environmental samples from the established SCT ICU previously housing these patients and staff (old ICU). While previous studies have longitudinally tracked surface and patient samples within an ICU, they have been limited in their ability to discern the impact of the facility built environment from the population of patients and healthcare workers in the facility. Here, the same patients and healthcare providers transitioned between the old and new buildings across the study period, allowing for a direct comparison between their ARO communities.

To track ARO transmission events between patients and ICU surfaces, we collected remnant fecal samples from patients in the SCT ICU who had laboratory studies ordered on fecal samples and isolates from positive blood cultures ordered as part of routine clinical care during the same collection period. From this unique collection of environmental and patient samples, we used selective microbiologic culturing and whole-genome sequencing (WGS) to identify AROs, assess antibiotic resistance, and track strains across time and location.

We found ARO contaminants were rare on most ICU surfaces but prevalent in sink drains in both ICUs, with the old ICU having significantly higher ARO burden in sink drains than the new ICU. AR Enterobacterales, which are frequently associated with fecal contamination, were rarely found on surfaces. In both ICUs, *Stenotrophomonas* spp. and *Pseudomonas* spp. were the two most frequently collected genera; however, different lineages dominated each ICU. *Stenotrophomonas maltophilia* strains formed months-long reservoirs in sink drains in the new ICU with no evidence of strains association with bloodstream infections during our study time period. In contrast, *Pseudomonas aeruginosa* strains formed persistent reservoirs for most of the year in the new ICU in multiple sink drains and showed evidence of shared strains across environmental samples and patient blood cultures. These results provide evidence that sink drains in the healthcare environment can serve as ARO reservoirs that are associated with human clinical infections.

## Methods

### Sample collections and culturing

Environmental and fecal samples received a non-human subjects determination by the Institutional Review Board (IRB) of Washington University (201712083). Blood culture clinical isolate collection was reviewed and approved by IRB (201901053) and by the Siteman Cancer Center Protocol Review and Monitoring Committee. We received IRB approval and Siteman Cancer approval for clinical isolates from patients. The IRB granted a waiver of informed consent for the collection of these specimens because they had been collected as part of routine clinical care. We sampled 6 SCT ICU (old ICU) rooms 3 times over the course of 1 month in the old building from December 2017 – January 2018. At each time point, nine surfaces were sampled using Eswab collections (Copan) pre-moistened with molecular water: the foam dispenser, the gown and glove storage area, the bedside rail, the nursing call button, the room floor, the light switch, the computer, the in-room sink handles, and the in-room sink drain. Three swabs were held together to simultaneously sample each surface. We also collected 2 samples of 15 mL in-room sink water directly from the faucet: 1 sample was collected immediately after turning the faucet on, and 1 sample was collected after allowing the water to run for 2 min.

We sampled 6 SCT ICU (new ICU) rooms and communal SCT ICU areas every other week for 5 months and then every month for 1 year in the new building for a total of 21 samplings (Fig. 1b). Samples were collected twice during the first week of sample collections in the new ICU building: the first after construction terminal clean and the second after custodial terminal clean. Both time points collected were before patients and staff had entered the ICU. At each time point, the same nine patient room surfaces as described above were sampled plus an additional 3 surfaces: the sofa from the patient room, the bathroom toilet from adjoining bathroom, and the sink drain from the adjoining bathroom. We also collected 15 mL of in-room sink water and bathroom sink water. At each time point, we also sampled four communal surfaces: the housekeeping closet drain, the family area floor, the soiled utility room drain, and the vending machine. For each time point in both buildings, we obtained remnant de-identified fecal specimens that had been submitted to the clinical microbiology laboratory for *C. difficile* testing from patients in the same unit as surface swab collection.

Eswab specimens from surfaces, water samples and fecal samples were cultured the same day of sampling. Eswab specimens were vortexed and 90 µL of eluate was used for culture inoculation per plate/test condition. For fecal specimens, 90 µL of specimen was used for culture inoculation. For water samplings, 100 µL of vortexed water sample was used for culturing. All samples were inoculated to each of the following culture medium: Sheep’s blood agar (Hardy), VRE chromID (bioMerieux), Spectra MRSA (Remel), HardyCHROM ESBL (Hardy), MacConkey agar with cefotaxime (Hardy), Cetrimide agar (Hardy), and Sabouraud dextrose + chloramphenicol (Hardy). Plates were incubated at 35 °C in an air incubator and incubated up to 48 h prior to discard if no growth (up to 7 days for sabouraud dextrose + chloramphenicol). Two colonies of each colony morphotype were subcultured and identified using matrix-assisted laser desorption/ionization time-of-flight mass spectrometry (MALD-TOF MS) with the VITEK MS system. All isolates recovered were stored at −80 °C in TSB with glycerol.

Isolates recovered from standard-of-care blood cultures during the same time frame of the surface sampling were recovered from frozen stocks in the clinical microbiology laboratory.

### Antimicrobial susceptibility testing

Antimicrobial susceptibility testing (AST) was performed using Kirby Bauer disk diffusion, interpreted according to CLSI standards^33^. AST was performed on gram negative bacilli using ampicillin, cefazolin, cefotetan, ceftazidime, ceftriaxone, cefepime, meropenem, ciprofloxacin, levofloxacin, piperacillin-tazobactam, ceftolozane-tazobactam, ceftazidime-avibactam, ampicillin-sulbactam, trimethoprim-sulfamethoxazole, gentamicin, amikacin, fosfomycin, colistin, aztreonam, doxycycline, minocycline, and nitrofurantoin and antimicrobials were interpreted/reported as appropriate for the specific species. We also performed a carbapenamase inactivation assay on all Enterobacterales and *Pseudomonas* isolates that were resistant or intermediate to meropenem or imipenem.

### Short read sequencing

Total genomic DNA was extracted from cultured isolates using the Bacteremia kit (Qiagen, Gernmantown, MD, USA) and DNA was quantified using the PicoGreen dsDNA assay (Thermo Fisher Scientific, Waltham, MA, USA). A total of 5 ng/µL was used as input for Illumina sequencing libraries with the Nextera kit (Illumina, San Diego, CA, USA). The libraries were pooled and sequenced on a NextSeq HighOutput platform (Illumina) to obtain 2x150bp reads. The reads were demultiplexed by barcode and had adapters removed with Trimmomatic^34^. Reads are available under BioProject PRJNA741123 (http://www.ncbi.nlm.nih.gov/bioproject/741123). Processed reads were assembled into draft genomes using SPAdes v3.11.0^35^. Assemblies were assessed for quality using Quast v3.2^36^ and checkM v1.0.13^37^. Assemblies were considered to have passed quality standards if completeness was greater than 90% and contamination was below 5%. We used Prokka on the assembled genomes to identify and annotate open reading frames^38^.

### Long read sequencing

Isolates were streaked from frozen stocks onto LB agar and allowed to grow at 37 °C for 48 h prior to extraction. Lawns were scraped from plates into nuclease free water. Genomic DNA was extracted using the bacteremia kit (Qiagen, Gernmantown, MD, USA), with the modification of limiting the vortex step to 2 min to preserve DNA fragment length. A total of 1 ug DNA from each isolate was used as input for library preparation using the Oxford Nanopore ligation sequencing kit and native barcode expansion kits (Oxford Nanopore Technologies, Oxford Science Park, OX4 4DQ, UK). Libraries were pooled and sequenced on a MinION flow cell (Oxford Nanopore Technologies, Oxford Science Park, OX4 4DQ, UK). Raw reads were preprocessed using Filtlong v0.2.0^39^ with parameters *–min_length 1000 –keep-percent 95 –target_bases 650000000*. Hybrid assemblies were created by assembling long read sequencing data in Flye v2.8.1^40^ and polished with short reads from Illumina sequencing^41^. Assemblies were assessed for quality using Quast v3.2^36^ and checkM v1.0.13^37^. Reads are available under BioProject PRJNA741123 (http://www.ncbi.nlm.nih.gov/bioproject/741123).

### Genomic taxonomic identification

Following draft assembly, we determined taxonomic identification by ANI, MASH, and MLST. Species were determined if the genome had >75% aligned bases and >95% ANI with the type genome. Assembled genomes were considered to be the same genomospecies if they had >95% pairwise match but no >95% match with a type genome. We compared all assembled genomes against all assembled genomes and all type genomes using dnadiff^42^. If no species were identified, we used Mash to determine genera by comparing assembled genomes against all NCBI reference genomes^43^. After all phages were removed, genera were considered to be the same as the hit/hits with the highest identity. MLST was determined using mlst v2.4^44,45^.

### Phylogenetic analyses

To create core genome alignments, the gff files produced by Prokka were used as input in Roary^46^. Roary alignments were used to create an approximate maximum likelihood tree with FastTree^47^. Branch length precision was rounded to 0.0001 substitutions per site. The output newick files were visualized and annotated with isolate source data using ggtree (R)^48,49^. Roary pangenome sequences were further annotated using EggNOG v5.0^50^.

### Isolate groupings based on SNP pairwise distances

Snippy v4.4.3^51^ was used to map forward and reverse reads for isolates to the type strain complete genome assembly and to call SNPs. To determine groups, we compared pairwise SNP distances between each isolate pairs of the same species. Isolates were grouped into perfectly reciprocal groups at every pairwise distance cutoff between isolates using igraph^52^. The SNP distance cutoff was set at the lowest SNP value where number of groups plateaued for many thousands of SNPs, indicating that the members of these groups are much more closely related to one another than other isolates.

### Antibiotic-resistant gene identification and analyses

We identified acquired antibiotic resistance mutations against aminoglycosides, amphenicols, β-lactam, folate pathway inhibitors, fosfomycin, macrolides/lincosamides/streptogramins, quinolones, rifamycin, tetracycline, and vancomycin using ResFinder^53^.

### Bayesian phylogenetic analysis of molecular sequences using BEAST 2

Group 1 isolates were long-read sequenced and quality filtered as described above, and the core genome alignment was constructed as above. The core genome alignment was composed of 5964 core genes out of 6986 total genes, which we used as input genes for our time-measured phylogenetic analysis in BEAST v2.6.5^54^. The core genome alignment was converted to a Nexus file using MEGA X^55^. We used BEAUti v2.6.5 from the BEAST v2.6.5^54^ software package to convert the Nexus file into a.xml file for input into BEAST. We chose to use the HKY site model because it allows for some flexibility in substitution rate for different types of substitutions, and catches most major biases^56^. We also used a strict clock model because our sequences are all from the same hospital within just over a year of each other, so we have no reason to suspect different substitution rates for different lineages^56^. Tip dates were determined as the number of days between each sample and the first sample collected. Model diagnostic information and parameter distribution were viewed using Tracer v1.7.2^57^. Individual trees were visualized using FigTree v1.4.4^58^ and the consensus tree was visualized using DensiTree v2.2.7^59^.

### Statistics and reproducibility

**Fig. 2:**
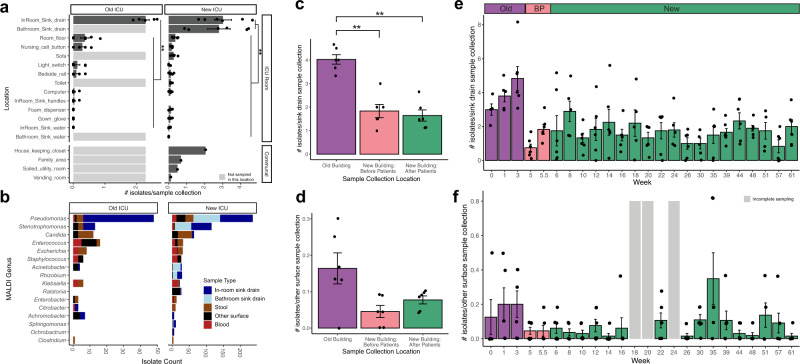
Variation in isolate collection location, identity, and timing across all sampling. Error bars indicate standard error of intensive care unit (ICU) rooms. ** indicates generalized linear mixed-modeling (GLMM) *p*-value <0.01. **a** In-room and bathroom sink drains have significantly more isolates per collection than other surface locations in both the old and new ICU buildings (*n* = 566 surface isolates). Locations in light gray were not collected in old ICU. **b** Genus of matrix-assisted laser desorption/ionization-time of flight mass spectrometry (MALDI-TOF) species identification of all collected isolates in both the new and old ICU. Other Surface includes all other surfaces that are not in-room or bathroom sink drain. **c** Variation in number of isolates collected per bathroom or in-room sink drain sample collection by building (excludes fecal and communal samples, *n* = 429). **d** Variation in number of isolates per other surface sample collection by building (excludes sink drain, fecal, and communal samples, *n* = 137). **e** Variation in number of isolates per bathroom or in-room sink drain sample collection for all time points, *n* = 429. **f** Variation in number of isolates collected per other surface sample collection across all time points (excludes sink drain, fecal, and communal samples, *n* = 137). Gray bars indicate weeks with incomplete sampling of surfaces. BP before patient and staff move-in.

Comparative statistics between old and new building samples were normalized by number of samplings. Generalized linear mixed models were used for significance testing, with Room and Week as random effects. In Fig. 2c, d, isolate frequencies were collapsed by Room and then averaged. Error bars indicate standard error. For all main text phylogenetic trees, branches with less than 80% bootstrap support were collapsed, and branches with 80–90% bootstrap support were labeled as such. Supplementary Figures containing phylogenetic trees (Supplementary Figs. 1c, d, 2, and 3a) have a minimum resolution of 0.00055.

### Reporting summary

Further information on research design is available in the Nature Research Reporting Summary linked to this article.

## Results

### AROs were collected and cultured from ICU surfaces, fecal samples, and clinical blood cultures in an old and new ICU

To test models of ARO reservoir colonization and transmission in a hospital built environment setting, we collected 1594 surface samples and 72 patient fecal samples at 24 time points from 6 ICU rooms in 2 buildings. Full metadata for 829 collected isolates has been included as Supplementary Data 1. The first building was the SCT ICU that was located in a well-established hospital building, the old ICU. The second was a newly constructed SCT ICU (new ICU); after construction was completed on the new ICU, the same staff and patients from the old ICU were all relocated to the new ICU. The old ICU rooms were sampled 3 times, with a week between samplings, during the final month of ICU occupancy (Fig. 1b). New ICU rooms were sampled twice (two days apart) after the completion of construction while the rooms were unoccupied, then once every other week for the first 5 months of patient and staff occupancy (*n* = 11 samplings), then once every month for the rest of the first year of occupancy (*n* = 8 samplings) (Fig. 1b). For both ICUs, we swabbed 10 high-touch ICU surfaces (with an additional 4 surfaces from attached bathrooms in new ICU rooms). We also obtained remnant fecal samples submitted for routine *Clostridioides difficile* testing as well as isolates recovered from standard-of-care blood cultures from patients in the ICU. We utilized selective microbiologic culture on surface and fecal samples to enrich for and culture AROs, including 1) organisms that form colonies on antibiotic media, which we later assessed for resistance phenotypes by antibiotic susceptibility testing (AST), and 2) organisms that are inherently resistant to antibiotics, including *Pseudomonas*, *Stenotrophomonas*, and *C. difficile*^60–62^. Results from AST can be found in Supplementary Data 2. Blood culture isolates were recovered in the clinical laboratory as part of routine clinical methods (i.e., not selectively cultured for ARO) and were retrospectively obtained for during 46 different weeks of the study spanning 61 weeks total. We recovered 566 AROs from surface environmental samples and 164 AROs from fecal samples, and we obtained 99 isolates from clinical blood cultures in the clinical microbiology laboratory.

### Sink drains had a high ARO burden compared to other ICU surfaces

To identify potential ARO surface reservoirs, we measured ARO burden (defined as number of different ARO isolates/morphotypes per samples collected) on different surfaces. Cultured bacteria were identified using VITEK MS matrix assisted laser desorption ionization-time of flight mass spectrometry (MALDI-TOF MS) (bioMerieux). ARO burden was significantly higher in sink drains than on other ICU room surfaces in both the old and new ICUs (Fig. 2a, GLMM: *p* < 0.001, marginal R^2^ = 0.942, conditional R^2^ = 0.945). All other ICU room surfaces had at least a 6-fold lower ARO burden (mean old ICU sink drains: 4.02 isolates/sample collection, mean old ICU other surfaces: 0–0.64 isolates/sample collection, mean new ICU sink drains: 1.59–1.72, mean new ICU other surfaces: 0–0.21). ICU sink water had low ARO burden (mean range of 0–0.02 isolates/sample collection) (Fig. 2a).

*Pseudomonas* was the most frequently detected genus, comprising 235/696 (33.8%) of all isolates cultured from the new ICU and 48/133 (36.1%) from the old ICU (Fig. 2b). The second most frequently identified genus was *Stenotrophomonas* (115/696 (16.5%) in the new ICU and 13/133 (9.8%) in the old ICU). Both genera were found primarily in sink drain samples (215/283 (76.0%) of *Pseudomonas* and 114/128 (89.1%) of *Stenotrophomonas*). Enterobacterales made up 77/696 (11.1%) and 20/133 (15.7%) of all isolates from the new and old ICUs, respectively, but only 7/97 (7.2%) were isolated from surface samples (Fig. 2b). *Candida* spp. isolates were identified in both the new ICU (64/696 (9.2%)) and old ICU (12/133 (9.0%)) with isolates primarily coming from fecal samples (50/76 (65.8%)) and room floor (10/76 (13.2%)). Gram positive AROs, including genera *Enterococcus*, *Staphylococcus*, and *Clostridium*, were found in both the new ICU (63/696 (9.0%)) and old ICU (23/133 (17.3%)). *Clostridium* was recovered from in fecal samples (*n* = 5). *Staphylococcus* and *Enterococcus* were found primarily in blood and fecal samples (52/81 (64.2%) of *Staphylococcus* and *Enterococcus*) and never found in sink drains. (Fig. 2b). These data suggest that in both buildings, sink drains are areas of substantial concern since they persistently yield cultures of *Pseudomonas* spp. and *Stenotrophomonas* spp., which both include strains capable of causing human infection^63,64^.

### ARO burden did not increase after patients and staff move in or over one year of sampling in new ICU

Next, we compared ARO burden across ICUs, patient and staff occupancy, and time points. Since there were large differences in ARO burden across surfaces, we separated environmental samples into 2 groups: sink drains and other (Fig. 2c, d). First, we compared ARO differences between the old ICU and new ICU before and after patient occupancy. We found ARO burden was higher in the old ICU than in the new ICU in sink drains (Fig. 2c, GLMM *p* < 0.001, R^2^ = 0.59) but not on other surfaces (Fig. 2d, GLMM *p* > 0.05, R^2^ = 0.07). Further, there was no difference in ARO burden before and after patient occupancy (Fig. 2c, d, GLMM p > 0.05). When we compared ARO burden in sink drains over time, we found no significant differences between the first week of collection after patients’ occupancy in the new ICU and any other time point collected (Fig. 2e, Wilcoxon signed-rank test *p* > 0.05). The same was true for other surface collections (Fig. 2f, Wilcoxon signed-rank test *p* > 0.05), although ARO burden for other surfaces had high variation across weeks (mean range 0.02–0.35). Together, this suggests that there were environmental-associated differences in ARO burden between the old and new ICUs, and that ARO burden did not change after patient occupancy in the new ICU nor significantly increase or decrease during 1 year of collections.

### No evidence of AR Enterobacterales reservoirs on surfaces in either ICU

To determine taxa-specific patterns in reservoir colonization, we performed WGS of Enterobacterales, *Pseudomonas*, and *Stenotrophomonas* isolates from environmental, fecal, and blood samples from both ICUs. AR Enterobacterales are some of the most feared AROs for HAIs^8^ and many are associated with human fecal colonization^65–67^. We collected 97 isolates from 4 genera of Enterobacterales: *Escherichia*, *Klebsiella*, *Citrobacter*, and *Enterobacter* (Supplementary Fig. 1A). Isolates were recovered primarily from fecal samples (45/97 (46.4%) of Enterobacterales) and from blood cultures (45/97 (46.4%) of Enterobacterales) (Supplementary Fig. 1A). *Escherichia coli* was the most frequently detected Enterobacterales species (37/97 (38.1%)), followed by *Klebsiella pneumoniae* (18/97 (18.6%)) (Supplementary Fig. 1A). Notably, from 1594 surface samples over 24 time points, there were only 7 instances of an Enterobacterales isolate being cultured from an ICU surface sample (Supplementary Fig. 1B). Of the 7 isolates, 2 were different morphotypes of *Citrobacter freundii* isolated from the same sample with high average nucleotide identity (ANI) (99.99%), suggesting closely-related organisms or morphovariants. Apart from those 2 *C. freundii* isolates, no 2 surface Enterobacterales were the same species and no 2 Enterobacterales were found on the same surface twice (Supplementary Fig. 1B). These data suggest AR Enterobacterales do not represent ARO reservoirs on any of the sampled ICU surfaces, despite being present in many patient fecal samples.

To determine within species isolate similarity, we compared strain genomes and antibiotic resistance profiles across the two most frequent Enterobacterales species: *E. coli* and *K. pneumoniae*. When we compared multi-locus sequence typing (MLST) profiles of *E. coli* isolates, we found one instance of shared sequence type (ST131) between a surface isolate and a blood or fecal isolate. In a core genome phylogenetic tree, we found no phylogenetic clustering based on isolate type or ICU, except for 3 different *E. coli* morphotype isolates all taken from the same fecal sample and sharing 99.98% ANI (Supplementary Fig. 1C). To determine if antibiotic resistance profiles vary by sample type or location, we determined phenotypic susceptibility and identified antibiotic resistance genes (ARGs) using Resfinder^68,69^. By Kirby Bauer disk diffusion, interpreted according to Clinical and Laboratory Standards Institute (CLSI) standards, 2/37 blood *E. coli* isolates were not resistant or intermediate resistant to any tested antibiotics. AST profiles varied across the *E. coli* isolates with isolates frequently resistant to ampicillin (23/37), cefazolin (20/37), ciprofloxacin (19/37), and levofloxacin (19/37) (Supplementary Fig. 1C). We found 9/37 *E. coli* isolates were resistant to cefepime, including the 1 surface isolate, and no *E. coli* isolates were resistant to meropenem. We found 24/37 *E. coli* isolates were resistant to multiple antibiotics with 20 isolates resistant to four or more drugs. *E. coli* isolates harbored various ARGs (Supplementary Fig. 1C), but ARG profile did not vary by sample type or location.

In *K. pneumoniae* isolates, we also found no phylogenetic clustering based on isolate type or ICU building (Supplementary Fig. 1D). Only one *K. pneumoniae* isolate, which was recovered from patient blood culture, demonstrated meropenem resistance, but it was negative for carbapenemase activity using the Carbapenem Inactivation Method^33^. 3/18 *K. pneumoniae* isolates were resistant to cefepime. 10/18 *K. pneumoniae* isolates were resistant to multiple drugs with 7 isolates resistant to four or more antibiotics. *fosA*, *oqxA*, and *oqxB* were found in a majority of isolates, 14/18 (78%), 17/18 (94%), and 17/18 (94%) respectively (Supplementary Fig. 1D). Together, these data show that while AR Enterobacterales were recovered from fecal specimens and can be a cause of blood stream infection in patients in the ICUs, these isolates were rarely found on surfaces, with no clear relationships between source of isolation and MLST, building, or antibiotic resistance. This suggests patient fecal contamination of sampled surfaces in these ICUs was rare and did not lead to ARO reservoir formation.

### Stenotrophomonas maltophilia strains are found persistently across one year of sampling in single ICU rooms

**Fig. 3:**
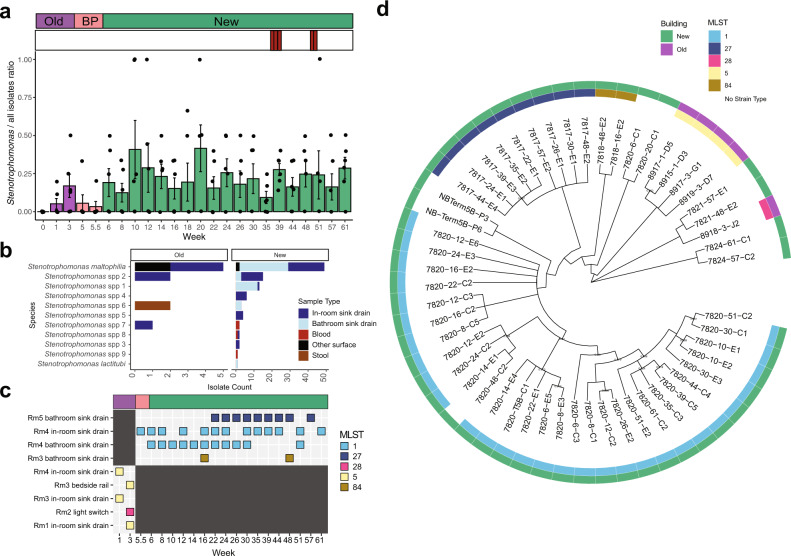
Timing, identity, and phylogenetics of *Stenotrophomonas* isolates. **a** Ratio of *Stenotrophomonas* isolates to all isolates across all time points (*n* = 128 *Stenotrophomonas* isolates). Error bars indicate standard error of intensive care unit (ICU) rooms. Red bars indicate collection timing of *Stenotrophomonas* blood culture isolates. **b** Identity of all collected *Stenotrophomonas* genomes by >95% average nucleotide identity (ANI) to reference genome by sample collection type (*n* = 128 isolates). Other Surface indicates all other surface/water genomes apart from in-room and bathroom sink drain. All genomes were identified as *Stenotrophomonas* by MASH. *Stenotrophomonas* various genomospecies includes all different genomospecies that did not share >95% ANI with a reference genome. **c** Time point mapping of shared *S. maltophilia* MLST groups by sample collection location. **d** Cladogram built from a core genome alignment of *S. maltophilia* genomes. Branches with less than 80% bootstrap support are collapsed. Branches with bootstrap values between 80–95% are labeled. BP before patient and staff move-in.

**Fig. 4:**
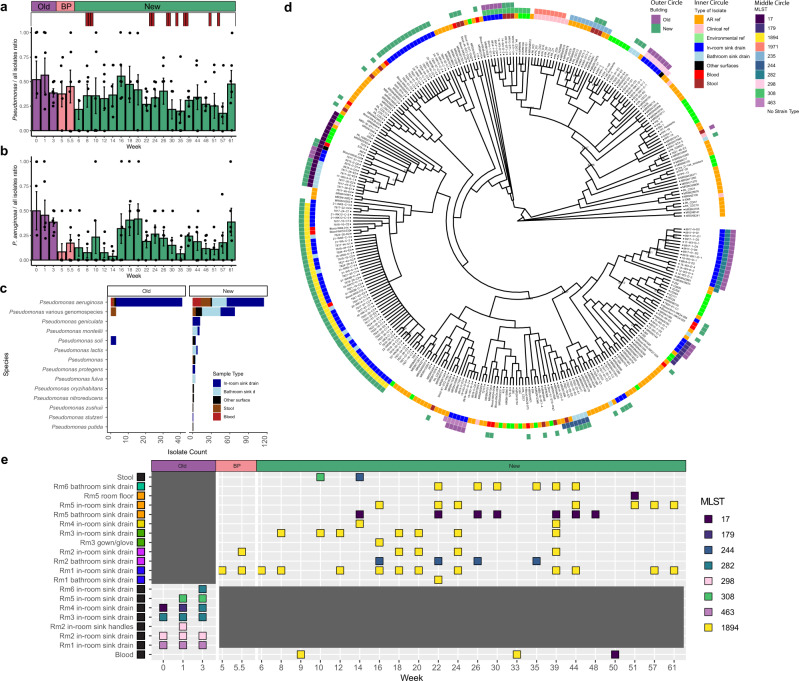
Timing, identity, and phylogenetics of *Pseudomonas* spp. isolates. **a** Ratio of *Pseudomonas* spp. to all isolates across all time points (*n* = 283 *Pseudomonas* isolates). Error bars indicate standard error. Red bars indicate collection timing of *Pseudomonas aeruginosa* blood culture isolates. **b** Ratio of *P. aeruginosa* to all isolates across all time points (*n* = 155 *P. aeruginosa* isolates). Error bars indicate standard error. **c** Identity of all collected *Pseudomonas* spp. genomes by >95% ANI to reference genome by sample collection type. Other indicates all other surface/water genomes apart from in-room and bathroom sink drain. All genomes were identified as *Pseudomonas* spp. by MASH. *Pseudomonas* various genomospecies includes all different genomospecies that did not share >95% ANI with a reference genome. **d** Cladogram from a core genome alignment of *P. aeruginosa* genomes. Branches with less than 80% bootstrap support are collapsed. Branches with bootstrap values between 80–95% are labeled. Reference *P. aeruginosa* genomes included antibiotic-resistant (AR) isolates, clinical isolates, and environmental isolates. Reference MLST is included if it shares a MLST with collected isolates. **e** Time point mapping of top 8 MLST *P. aeruginosa* groups by sample collection location. BP before patient and staff move-in.

While *S. maltophilia* is predominantly found in environmental water sources, the species is an emerging pathogen associated with HAIs, particularly in immunocompromised patients; these infections are associated with substantial case fatality rates^64^, primarily because of the intrinsic antimicrobial resistance of this microorganism and the vulnerable patient population that it affects. *Stenotrophomonas* spp. were isolated from every week sampled, except for the first week of collection in the old ICU, although the ratio of *Stenotrophomonas* isolates to all collected isolates varied over time (Fig. 3a). Among 128 isolates identified as *S. maltophilia* by MALDI-TOF MS, ANI species identification and MASH genus identification typed them as 54 *S. maltophilia* isolates, 1 *S. lactiubi*, and 53 *Stenotrophomonas* spp. (not otherwise specified) in 9 genomospecies groupings (Fig. 4b). When we compared MLST and core genome phylogeny of *S. maltophilia* isolates, we found that sequence type and phylogenetic clades were not shared across ICUs (Fig. 3c, d, Supplementary Fig. 2). Only two sequence types were identified on the same respective surface over multiple weeks, suggesting that these surfaces acted as reservoirs (Fig. 3c, blue sequence types). *S. maltophilia* of ST27 was found 9 times over 35 weeks, and *S. maltophilia* of ST1 was found 13 times over the course of a year (56 weeks), including before patient and staff occupancy (Fig. 3c). Both sequence types remained in the same room, with no evidence of crossover between rooms in the new ICU (Fig. 3c). Phenotypic susceptibly demonstrated no isolates with trimethoprim-sulfamethoxazole or minocycline resistance, one isolate with levofloxacin resistance, and 34/54 isolates with colistin resistance.

### *P. aeruginosa* were diverse and found persistently across one year of sampling and in all 6 new ICU rooms

While commonly found in the environment, *Pseudomonas* spp. have a long history of causing HAIs^60,70–73^. Studies have shown that *P. aeruginosa* reservoirs established in hospital built environments can lead to infections and outbreaks^74–78^. However, it is unclear when these reservoirs became established, relative to patient or staff occupancy of the healthcare environment, and how pervasively *Pseudomonas spp*. may colonize ICU surfaces. We recovered more *Pseudomonas* spp. isolates than any other genus during our collections (Fig. 2b). MALDI-TOF MS identified 283 *Pseudomonas* spp. isolates. *Pseudomonas* spp., and particularly *P. aeruginosa*, isolates were collected at every time point in the study period, including before patient occupancy (Fig. 4a, b). After ANI species identification and MASH genus identification, we found 155 *P. aeruginosa* isolates, 71 *Pseudomonas* spp. isolates in 13 genomospecies groupings, and 54 isolates from other *Pseudomonas* species (Fig. 4c). Most *P. aeruginosa* isolates were from surface samples (80%); 11% were from fecal samples; and 9% were from blood cultures (Fig. 4c). We did not find overlap between any other *Pseudomonas* spp. isolates from patient blood cultures and environmental samples.

When we compared MLST and core genome phylogeny of *P. aeruginosa* isolates, we find that isolates from different ICUs fall into different clades and strain types (Fig. 4d, e, Supplementary Fig. 3). To understand the genomic context of *P. aeruginosa* isolates, we compared the genomes of isolates recovered from surface and patient sampling with 172 reference *P. aeruginosa* genomes downloaded from NCBI (Supplementary Data 3). Reference genomes were phylogenetically diverse and fell into 3 categories: (i) isolates from clinical infections, (ii) AR isolates from the CDC with known antibiotic resistance, and (iii) environmental isolates that had been collected from water and waste projects. The isolates we collected from both the old and new ICUs spanned most of the diversity of *P. aeruginosa* with no distinct clustering between collected ICU surface isolates and environmental, clinical, or AR isolates (Fig. 4d). Although there were no distinct clades based on isolate building or surface source, we do find that our isolates form a number of clades with highly-related surface isolates (Fig. 4d). These frequently corresponded with sequence type. There were two cases of overlap in sequence type between the old and the new building (Fig. 4e). ST17 was found in sink drains in both the old and new ICU and found in a blood culture in the new ICU. ST170 was found in surface samples in the old ICU and a patient fecal sample in the new ICU (Fig. 4e). Notably, *P. aeruginosa* of ST1894 was recovered from the same sink drain beginning before patient occupancy and continuing through for the full year of collection in the new ICU. This repeated isolation of ST1894 suggests that it may have established a continuous reservoir in this room in the new ICU. Furthermore, isolates of *P. aeruginosa* of ST1894 were also recovered from sink drains in all 6 sampled ICU rooms and were found across 5 or more time points in 5/6 sampled ICU rooms (Fig. 4e), suggesting this colonization and persistence is more widespread. Finally, we found that 3 blood culture isolates (3.7% of all blood culture isolates tested) also belonged to ST1894, which prompted a higher resolution comparative analysis of all ST1894 strains, due to its potential to contaminate the environment and be associated with bloodstream infections.

### Antibiotic resistance in *P. aeruginosa* isolates varies between the two ICUs

**Fig. 5:**
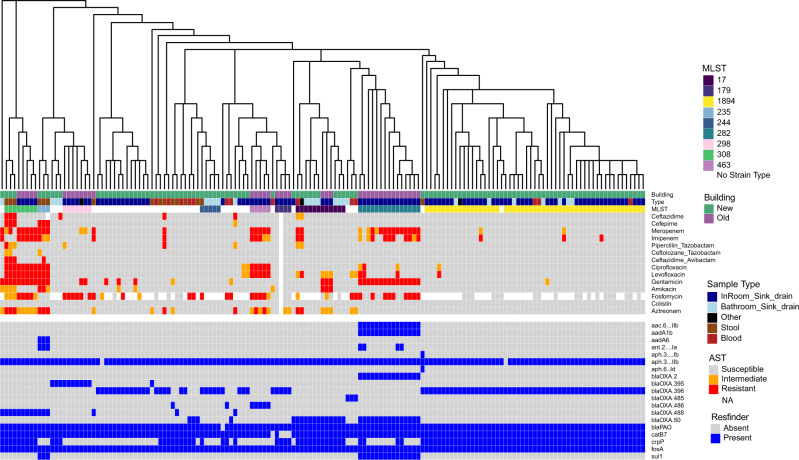
Phenotypic and genotypic antibiotic resistance of collected *P. aeruginosa* isolates. Phylogenetic tree is from a core genome alignment. Phenotypic resistance determined by antibiotic susceptibility testing (AST). Genotypic resistance determined by Resfinder^53^.

To determine if antibiotic resistance profiles vary by location, we determined phenotypic susceptibility using antibiotic susceptibility testing (AST) and identified ARGs in assembled genomes using Resfinder^53^. *P. aeruginosa* are defined as AROs because of their intrinsic resistance to many aminoglycosides, tetracyclines, β-lactams, and quinolones;^60,79^ we performed ASTs for 14 antibiotics for all *Pseudomonas* isolates to measure acquired resistances to β-lactams, cephalosporins, carbapenems, penicillins, fluoroquinolones, aminoglycosides, and polymyxins. AST profiles were similar across *P. aeruginosa* of the same sequence type (Fig. 5). *P. aeruginosa* isolates of ST1894 were largely not resistant to the antibiotics tested*. P. aeruginosa* isolates of ST282 were resistant to meropenem (11/15) and gentamicin (15/15). *P. aeruginosa* isolates of ST308 were resistant to meropenem (6/8), imipenem (5/8), ciprofloxacin (8/8), levofloxacin (8/8), and gentamicin (8/8). As different sequence types dominated the different ICUs and resistance profiles were similar across sequence types, we found trends in resistance to be different between the two ICUs with isolates from the old ICU having a higher percentage of resistance to meropenem and imipenem than *P. aeruginosa* isolates from the new ICU (new ICU: 7% imipenem, 7% meropenem and old ICU: 40% imipenem, 55% meropenem) (Fig. 5).

Much like the AST profiles, the ARG profiles also appeared to be linked to sequence type (Fig. 5). Nearly all *P. aeruginosa* isolates carried the resistance genes *aph(3’)-IIb* (153/155), *blaPAO* (154/155), *catB7* (151/155), and *fosA* (155/155). Isolates from ST282 were the only identified to contain the aminoglycoside resistance genes *aac(6’)-IIb* (15/15) and *aadA1b* (15/15), which could explain the phenotypic resistance to gentamicin. All isolates from ST 1894 carried the β-lactam resistance gene *blaOXA-396* (52/52), while it was less common in other sequence types (35/103). *P. aeruginosa* is also capable of developing resistance to carbapenems (meropenem, imipenem), fluoroquinolones (ciprofloxacin, levofloxacin), and aminoglycosides (gentamicin) by chromosomal point mutations, rather than acquisition of ARGs^80,81^.

### *P. aeruginosa* Group 1 strain was found across 1 year of sampling and in both environmental and patient samples

**Fig. 6:**
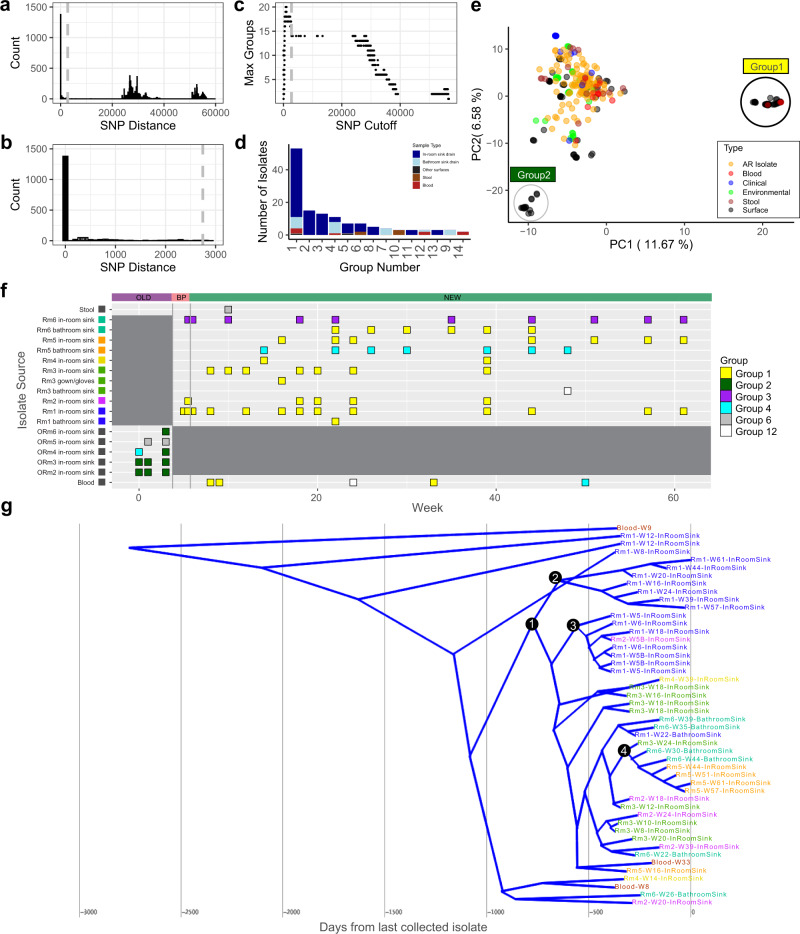
Highly-related genomic groups of *P. aeruginosa* across locations and time. **a** Histogram of pairwise single nucleotide polymorphism (SNP) distances between *P. aeruginosa* genomes indicate three modes of pairwise distances. The first corresponds to highly-related genomic groups. We define group SNP threshold as pairwise distances that fall before 2743 (gray dashed line). **b** Zoomed in histogram of pairwise SNP distances between *P. aeruginosa* genome with a cut off at 3000 SNPs show only highly-related genomic groups. **c** Max groupings by SNP cut off show pairwise groups plateau at 2743 SNPs. **d** Number of isolates per highly-related genomic group. Other surfaces includes all other surfaces that are not in-room or bathroom sink drain. **e** First two components of principal component analysis (PCA) of the accessory genome of all *P. aeruginosa* genomes. Black circle encloses all Group 1 *P. aeruginosa* genomes. Gray circle encloses all Group 2 *P. aeruginosa* genomes. **f** Time point mapping of top 4 *P. aeruginosa* highly-related groups and highly-related groups that shared isolates between patient and surface samplings. **g** Time-measured phylogenetic analysis consensus tree of *n* = 48 Group 1 *P. aeruginosa* isolates depicted using DensiTree v2.2.7^59^. Nodes labeled with black circles. Node 1 marks the main clade with a time since most recent common ancestor (TMRCA) of 778 days. BP before patient and staff move-in.

While MLST has been used previously to describe strains and outbreaks, it is limited to a small number of genes or alleles and does not enable genome-resolved understanding of strain relatedness. Accordingly, here we utilized WGS data for each *P. aeruginosa* isolate to perform an in-depth analysis of similarity across genomes. We calculated pairwise SNP distances by mapping quality filtered short-reads from all *P. aeruginosa* isolates to a high-quality, long-read sequencing-assembled genome of the first temporal occurrence of ST1894, with a mean of 89.8% of reads mapped to the genome. We then used a grouping technique on *P. aeruginosa* isolates to find fully reciprocal groups^82^. We compared pairwise SNP distances between *P. aeruginosa* isolate pairs and iterated through each unique SNP distance cutoff to filter the isolate pairwise network list (Fig. 6a, b). For each SNP cutoff, we determined the number of complete subgraph groups, defined by each node in the group was connected to every other node in the group, and isolates per group. The number of *P. aeruginosa* groups rose initially from 3 to 18 groups as SNP distances increased from 0 to 377 SNPs. After a peak at 756 SNPs with 20 groups, the total number of groups slowly decreased to a plateau of 14 groups at 2743 SNPs (Fig. 6c). From this, we determined an appropriate SNP cutoff that separated closely-related isolates from other groups was 2743. Using this definition, *P. aeruginosa* isolates fell into 14 groups, with the largest group (Group 1) including 53 isolates (Fig. 6d). Only three groups had isolates that spanned patient and environmental isolates: Group 1, Group 6, and Group 12. Group 1 had no more than 11 SNPs between isolates and included isolates from blood cultures and environmental samples. 52/53 of the isolates in Group 1 were ST1894, and the remaining isolate was unidentified but had 5/6 alleles identical to ST1894. The isolates in this group persistently and pervasively colonized new ICU sink drains and were cultured from sink drains 49 times across 56 weeks (Fig. 6f). Aside from sink drains, Group 1 was also found in 3 patient blood cultures, 1 of which was isolated from a different ward in the same building (Fig. 6f). 1 isolate from Group 1 was isolated from the gown and glove personal protective equipment box located just outside the room. All isolates within this group were within 11 short-read SNPs of each other. Group 2 (ST17) was found once in a sink drain in the old ICU, 7 times in the bathroom sink drain of Room 5 in the new ICU, and once in a blood culture isolate. Group 12 (ST241) was found once in a sink drain in the new ICU, and once in a blood culture isolate. This highlights 3 instances where a sink drain isolate was found within the same genome-resolved group as a blood culture isolate of a patient in the ICU.

When we compared the accessory genomes of our cultured isolates and reference *P. aeruginosa* genomes, we found a mean of 4018 (range 3221–5003) accessory genes per genome. Group 1 isolates have a mean of 3947 (range 3885–4022) accessory genes, suggesting average accessory genome size. To compare variation in accessory genomes across *P. aeruginosa* we used a principal component analysis (PCA). We found distinct clustering between Group 1 isolates and the rest of the *P. aeruginosa* isolates (PERMANOVA: *p* < 0.001) (Fig. 6e). There were 36 accessory genes with high loading scores on PC1 that are unique to Group 1, of which only 7 could be characterized by EggNOG (Supplementary Data 4, Supplementary Data 5)^50^.

### *P. aeruginosa* Group 1 isolate lineages clustered by room

SNP analysis from short reads allows us to accurately estimate genomic relatedness and group highly-related genomes, but obtaining fully-resolved genomes is necessary to identify transmission and reservoir persistence in sink drains. Our short-read data indicated <11 SNPs between Group 1 isolates, which is well within previously established probable transmission for *P. aeruginosa*^83^. Indeed, our phylogenomic tree building grouped isolates similarly to this analysis (Fig. 4d, Supplementary Fig. 3). To investigate reservoir formation of *P. aeruginosa* Group 1 isolates over time at higher genomic resolution, we obtained long-read sequencing data for the 53 isolates in Group 1. We created hybrid assemblies of each genome (assemblies had between 1–11 and an average of 4.32 contigs) and found the core genome to consist of 4863 genes out of 9714 total genes. By examining the accessory genome, we identified 4 additional isolates that were responsible for a large portion of the accessory genome and removed them from analysis, as they were unlikely to be part of the same lineage. The remaining 49 Group 1 isolates consisted of 5964 core genes from 6986 total genes.

In our time-measured phylogenetic analysis using BEAST^54^, we created a consensus tree of estimated time since most recent common ancestor (TMRCA) using the Group 1 core genome (Supplementary Fig. 4). As we do not have isolates collected this far back, confidence in branch divisions is low (Supplementary Fig. 5) and the TMRCA of 8034 days was largely driven by one isolate, which was removed from further analysis. The remaining isolates formed a consensus tree (Fig. 6g) with a TMRCA of 2752 days with a 95% highest posterior density interval (HPD) of 1523–4362 days. 38/48 of these isolates were taken from the in-room sink drains; 7 were found in the sink drains from the attached bathroom; and 3 were isolated from blood infections.

The majority (40/48) of these isolates are contained under Node 1 and have a TMRCA of 778 days with a 95% HPD of 488–1122 days. This clade displays 3 unique evolutionary patterns. Descending from Node 2, eight isolates cluster together. 7/8 of these isolates were taken from the in-room sink drains in Room 1 within the first 2 weeks of the study, suggesting the diversity shown represents heterogeneity within a given sink rather than in-room evolution. We also found two likely instances of within room evolution, marked by Nodes 3 and 4. These isolates branch off from one lineage as time progresses, suggesting an evolving, single reservoir. Outside of these main features, the remaining isolates under Node 1 were from mixed rooms and collection weeks with low confidence in the branching (Supplementary Fig. 4). The commingling and low confidence in branching suggests strain exchange between rooms of a common pandemic strain throughout the sampling period.

Interestingly, one of the blood isolates clustered with an environmental isolate within the clade under Node 1, but the other two were further removed from the bulk of Group 1. While all isolates were collected from patients in this ward at some point during their stay, this isolate was collected two days before the patient moved into Room 5. The other two blood isolates were further removed from Node 1 environmental isolates. However, there are possibly different evolutionary pressures within each sample type, which could drive different mutation rates. The overall close relation of the blood and surface isolates implies direct correlation within the duration of the study period and potentially presents a great risk to patient safety.

## Discussion

The process of ARO reservoir colonization of the hospital built environment is dependent on complex interactions, and transmission events to vulnerable patients are not well understood^27,84,85^. In this study we investigated the microbiologic changes in a new SCT ICU before and after patient or staff occupancy and tracked ARO strains cross ICU surfaces and patients. We identified a mechanism of ARO colonization development that occurred prior to patient or staff move-in, which could promote the necessity of future surveillance investigations. We compared these colonization patterns to equivalent microbial sampling in the corresponding old SCT ICU during its final month of occupancy, before patients and staff moved from there to the new ICU, to obtain a unique comparison of distinct hospital built environments following relocation of patients and hospital staff. We found ARO reservoirs were rare on most hospital surfaces apart from sink drains. Non-sink drain surfaces showed no difference in ARO burden between the two buildings, but sink drains in the old ICU had a significantly higher burden than those in the new ICU. Further, reservoir colonization and transmission varied by taxa and between buildings, with evidence in *P. aeruginosa* of shared strains across multiple sinks and human clinical infections in the new ICU.

Recent studies have focused on better understanding and characterizing the hospital microbiome using metagenomics^11–15^. These characterizations find correlations between samples of hospital surfaces, patients, and staff, particularly in skin- and gut-associated taxa such as Enterobacterales and *Staphylococcus*, suggesting the microbiomes of humans and the hospital built environment influence each other^11–15^. Further, strain tracking using metagenomic analyses indicates that similar strains may be present on surfaces over time, suggesting potential reservoir colonization on surfaces^11,13^. Building on these studies, we focused on high-resolution, temporal, genomic and phenotypic investigation of viable AR strains which colonize or infect surfaces and patients in ICUs. We found that AROs isolated from patient stools were rarely found on ICU surfaces, and with the exception of sink drains, we do not find persistent reservoir colonization of most ICU surfaces. In contrast we found multiple instances of ARO reservoir colonization of ICU sink drains, with highly-related strains of these AROs also recovered from patient blood cultures.

We found AROs more frequently in sink drains in the old ICU compared to the new ICU. There are many possible reasons for these differences, including: building material and layout differences, water sources, natural history, and extended time for establishment and accumulation of AROs^86–89^. In the new ICU, AROs were found before patient or staff occupancy, and ARO burden in both sink drains and other surfaces did not significantly change after 1 year of patient occupancy. This baseline level of ARO burden in an ICU suggests that patients are not the primary source of AROs found on surfaces nor do they cause significant increases in ARO burden during the first year of ICU establishment. Further, these results have important implications for remediation strategies that involve removing or rebuilding infrastructure and suggests such strategies may not always be successful.

When comparing ICU room surfaces, we found AROs more frequently on sink drains and rarely on any other ICU room surface. While studies in low to medium income countries have found high ARO burdens on hospital surfaces, our results are consistent with other studies in the United States (US) that have found low ARO burden on ICU surfaces and high ARO burden in sink drains^82,90^. While it is possible that our sampling methods may miss some AROs, the sparsity and inconsistency of AROs on surfaces suggests that most surfaces other than sink drains are not acting as persistent reservoirs for AROs. It may also be that some AROs do not survive well on dry ICU surfaces where they cannot easily form biofilms^88,90^. However, other studies have found ARO colonization on these types of surfaces for long periods, suggesting that colonization is possible^82,91–93^. Instead, high standards of cleaning, self-disinfecting equipment, and special training in high income countries such as the US may be effective at removing and limiting ARO reservoirs on most commonly-touched surfaces^91,94,95^. While national standards and studies have suggested protocols for cleaning many hospital surfaces^95^, there are no standardized protocols for cleaning sink drains. This may lead to variable and inconsistent decontamination of these areas compared to other commonly-touched surface areas. Further, sink drains are often difficult to clean as liquid disinfectant is less effective when poured down the drain without coating the drain surface, and the drains are often covered by a drain cover and cannot easily be wiped down or scrubbed^96,97^.

Reservoir colonization by AROs in sink drains appears to be specific to particular taxa. While we cultured a wide diversity of AROs from sink drains, only two species had strains that formed reservoirs in sink drains: *S. maltophilia* and *P. aeruginosa*. These results corroborate previous work identifying *Pseudomonas* spp. and *Stenotrophomonas* spp. as capable of long-term colonization of sink drains^74–77^. In contrast, we did not find evidence of persistent colonization of sink drains by Enterobacterales species, which have commonly been associated with hospital built environment outbreaks^17,28,88,98–100^. It is possible these organisms were present but weren’t isolated because they were not resistant to the antibiotics used in selective culturing.

*S. maltophilia* is an environmental organism that is emerging as a serious concern for HAIs and other infections^64^. For our purposes, we defined reservoirs to mean surfaces where at least 2 isolates from the same sequence type were isolated from consecutive samplings. We found reservoirs of *S. maltophilia* in at least 3 sink drains. *S. maltophilia* ST1 established reservoirs in two surfaces of the same ICU room, suggesting a similar source or the spread of one strain type to a different location. However, we find little evidence of strain transfer to sink drains in other rooms in the same ICU, and no evidence of transmission to patients. In fact, while we found 3 *Stenotrophomonas* isolates in blood cultures, when we used ANI to identify species, none of these were identified as *S. maltophilia*. This may have broad clinical applications as poor identification of blood isolates could potentially lead to inappropriate treatment. However, even though we find no evidence of transmission of *S. maltophilia* sink strains to patients, since *S. maltophilia* has been shown to be a pathogen in immunocompromised patients, it is still important to identify methods to remove sink drain reservoirs of these organisms.

*P. aeruginosa* has long been characterized as an opportunistic pathogen that inhabits environmental sources, particularly water sources, as well as the human gut^60,70–73^. When compared to diverse *P. aeruginosa* genomes from other studies, we found no distinct clustering with environmental or clinical isolates, suggesting that our isolates are not coming from a strictly environmental strain pool. Instead, the strains we characterized were phylogenetically diverse, indicating that the adaptations necessary to survive in sink drains in the ICU are not restricted to a single clade. Further, there was limited apparent transfer of surface isolates between ICUs as patients and staff moved from one location to the other, as strains were unique between surfaces in the old and new ICU.

Remarkably, the genomic diversity of *P. aeruginosa* isolates from ST1894 in sink drains was incredibly low, even after one year or sampling*. P. aeruginosa* ST1894 was first described in 2014 in a cystic fibrosis patient in Spain (Isolate RC19, id:2398)^44^. Since only the MLST was done for this isolate and not WGS, it is impossible to determine if this isolate and our ST1894 isolates have similar ancestry. The ST1894 isolates from our study are not only capable of surviving well in sink drains but also of colonizing multiple sink drains; our collection scheme documents Group 1 *P. aeruginosa* of ST1894 first being cultured from a single room, but after 17 weeks of sample collection, was found in all six ICU rooms samples. Our initial short read-based WGS approach provided the resolution to cluster isolates into groups based on whole-genome SNP distances, which has been the mainstay for transmission dynamics up until this point^83,101,102^. However, our long-read sequencing analysis elucidated the more nuanced relationships necessary for transmission and reservoir colonization dynamics. Specifically, our phylogenetic analysis with high-quality hybrid assemblies indicates key cases of a ST1894 strain inhabiting one sink drain before patients move into the hospital, and then spreading and exchanging between all rooms sampled. Our sampling illuminated the diversity and evolution of this lineage across time and space during the course of the study period. We also found evidence of 3 instances where this strain was found in blood cultures from hospitalized patients, highlighting ST1894 as an urgent threat to this healthcare facility and associated immunocompromised patients. The bias for these isolates originating from the in-room sink drains rather than the bathroom sink drains also suggests that the water source system, which is common to all drains, is not a likely source of reservoir contamination. This, in association with the patient sample that was collected outside the sampling ward, lead us to suspect this strain may be more widespread in this healthcare system than our sampling area. Our genomic analyses indicate that *P. aeruginosa* ST1894 has a very unique accessory genome compared to other *P. aeruginosa*, thus leaving a long list of candidate genes that might explain its prevalence in sink drains. Further investigation into these genes and other similar strains will help us better understand the genomic evolution that might have allowed for its environmental pathogenicity.

Globally, antibiotic resistance in *P. aeruginosa* isolates is a growing concern, with infection mortality rates of 33–71% in carbapenem-resistant infections^81^. *P. aeruginosa* is capable of both intrinsic chromosomal modifications and acquisition of mobile ARGs that encode resistance to all classes of antibiotics currently used in *P. aeruginosa* treatment. However, carbapenem resistance in *P. aeruginosa* has only been acquired through the acquisition of mobile ARGs, most commonly metallo-$\beta$-lactamases (MBLs) and are typically encoded on plasmids, integrons, and mobile cassettes^81^. In general, carbapenem resistance was rare in *P. aeruginosa* isolates collected in the new ICU, while it was common in *P. aeruginosa* isolates collected in the old ICU. *P. aeruginosa* ST1894 was generally susceptible to the suite of antibiotics we tested against, with only 2 instances of resistance observed. Fortunately, this means there are currently a number of viable antibiotic treatment options against the existing reservoirs of ST1894 in our healthcare system. However, the presence of other *Pseudomonas spp*. with much higher AR burdens in this same hospital environment, and the known ease of resistance transmission in *Pseudomonas spp*., emphasizes the risk that this widely disseminated ST1894 reservoir could evolve into a greater ARO threat.

Despite our success in identifying multiple reservoirs with our current methods, it is plausible that we are under sampling the genomic diversity and persistent colonization through the cross section of time points sampled. For example, in our identification of reservoirs by Group 1 of *P. aeruginosa* (Fig. 6f), we believe the strain was likely still present even when it appears to skip certain weeks. Even with selection of multiple isolates per selective plate, further work could improve these methods, such as a metagenomics based approach, and reveal additional reservoirs.

It is intriguing that many AROs were found in sink drains even prior to patient relocation to this unit. Previous work has suggested sources of contamination such as patient or hospital staff carriage of *P. aeruginosa*^103^, or diffusion through water pipes^103,104^, but these don’t address contamination identified prior to patient or staff move-in. Other studies have identified water contamination as a potential source^75,76,105^, but our sampling did not indicate water as the source of these AROs. Further research is necessary to understand the origins of the strains. Regardless of their origins, these findings highlight the need for a more thorough decontamination procedures, both during the terminal clean and regular operation of ICU facilities.

In conclusion, our investigation of ARO reservoirs allowed us to assess and compare models of colonization and transmission in an old and new hospital built environment with the same patient and staff populations, including before and after patient or staff occupancy. Our approach of selective microbiologic culture combined with WGS analyses provide for a detailed analysis of ARO variation across one year of sampling in an SCT ICU. Together these data provide a high-resolution characterization of AROs in the hospital built environment, highlighting that SCT ICU sink drains are a major reservoir for AROs with direct links to patient infections. Most pressingly, the surprisingly rapid development of *P. aeruginosa* colonization and association with patient infections emphasizes the need for future work to decrease the spread of AROs in hospital built environments, completed by efforts towards decolonizing and eliminating sink drain ARO reservoirs.

## Supplementary information

Description of Additional Supplementary Files

Supplementary Data 1

Supplementary Data 2

Supplementary Data 3

Supplementary Data 4

Supplementary Data 5

Reporting Summary

Supplementary Information

## Data Availability

All genomic reads generated during and/or analyzed during the current study are available under BioProject PRJNA741123. Other source data for the main figures can be found in Supplementary Data 1–5.
