# Supplementary material for: Antibiotic-resistant organisms establish reservoirs in new hospital built environments and are related to patient blood infection isolates
Source: Commun Med (Lond). 2022 Jun 1;2:62. doi: 10.1038/s43856-022-00124-5 (PMC9160058; doi:10.1038/s43856-022-00124-5)
Supplement: Supplementary file 1 — Description of Additional Supplementary Files [file 43856_2022_124_MOESM1_ESM.pdf]

Description of Additional Supplementary Files

File Name: Supplementary Data 1

Description: Full metadata for 829 collected isolates.

File Name: Supplementary Data 2

Description: AST profile results for tested isolates.

File Name: Supplementary Data 3

Description: 172 reference P. aeruginosa genomes downloaded from NCBI.

File Name: Supplementary Data 4

Description: Functional annotation of accessory genes from P. aeruginosa isolates as output by EggNOG.

File Name: Supplementary Data 5

Description: PC loading scores of accessory genes from P. aeruginosa isolates.
