# Supplementary material for: Antibiotic-resistant organisms establish reservoirs in new hospital built environments and are related to patient blood infection isolates
Source: Commun Med (Lond). 2022 Jun 1;2:62. doi: 10.1038/s43856-022-00124-5 (PMC9160058; doi:10.1038/s43856-022-00124-5)
Supplement: Supplementary file 7 — Reporting Summary [file 43856_2022_124_MOESM7_ESM.pdf]

nature portfolio

Corresponding author(s): Erin P Newcomer

Last updated by author(s): Apr 6, 2022

Reporting Summary

Nature Portfolio wishes to improve the reproducibility of the work that we publish. This form provides structure for consistency and transparency in reporting. For further information on Nature Portfolio policies, see our Editorial Policies and the Editorial Policy Checklist.

Statistics

For all statistical analyses, confirm that the following items are present in the figure legend, table legend, main text, or Methods section.

n/a	Confirmed	
☐	☒	The exact sample size (*n*) for each experimental group/condition, given as a discrete number and unit of measurement
☐	☒	A statement on whether measurements were taken from distinct samples or whether the same sample was measured repeatedly
☐	☒	The statistical test(s) used AND whether they are one- or two-sided<br>*Only common tests should be described solely by name; describe more complex techniques in the Methods section.*
☐	☒	A description of all covariates tested
☐	☒	A description of any assumptions or corrections, such as tests of normality and adjustment for multiple comparisons
☐	☒	A full description of the statistical parameters including central tendency (e.g. means) or other basic estimates (e.g. regression coefficient) AND variation (e.g. standard deviation) or associated estimates of uncertainty (e.g. confidence intervals)
☐	☒	For null hypothesis testing, the test statistic (e.g. *F*, *t*, *r*) with confidence intervals, effect sizes, degrees of freedom and *P* value noted<br>*Give P values as exact values whenever suitable.*
☐	☒	For Bayesian analysis, information on the choice of priors and Markov chain Monte Carlo settings
☒	☐	For hierarchical and complex designs, identification of the appropriate level for tests and full reporting of outcomes
☐	☒	Estimates of effect sizes (e.g. Cohen's *d*, Pearson's *r*), indicating how they were calculated

Our web collection on statistics for biologists contains articles on many of the points above.

Software and code

Policy information about availability of computer code

Data collection	No software.
Data analysis	The reads were demultiplexed by barcode, had adapters removed with Trimmomatic. Processed reads were assembled into draft genomes using SPAdes v3.11.0. Assemblies were assessed for quality using Quast v3.2 and checkM v1.0.13. We used Prokka on the assembled genomes to identify and annotate open reading frames. For long read sequencing data, raw reads were preprocessed using Filtlong v0.2.0. Hybrid assemblies were created by assembling long read sequencing data in Flye v2.8.1 and polished with short reads from Illumina sequencing. Assemblies were assessed for quality using Quast v3.2 and checkM v1.0.13. We compared all assembled genomes against all assembled genomes and all type genomes using dnadiff. If no species were identified, we used Mash to determine genera by comparing assembled genomes against all NCBI reference genomes. MLST was determined using mlst v2.4. To create core genome alignments, the gff files produced by Prokka were used as input in Roary. Roary alignments were used to create an approximate maximum likelihood tree with FastTree. Roary pangenome sequences were further annotated using EggNOG v5.0. Snippy v4.4.3 was used to map forward and reverse reads for isolates to the type strain complete genome assembly and to call SNPs. Isolates were grouped into perfectly reciprocal groups at every pairwise distance cutoff between isolates using igraph. We identified acquired antibiotic resistance mutations using ResFinder. We conducted our time-measured phylogenetic analysis using BEAST v2.6.5. The core genome alignment was converted to a Nexus file using MEGA X. We used BEAUti v2.6.5 from the BEAST v2.6.5 software package to convert the Nexus file into a .xml file for input into BEAST. Model diagnostic information and parameter distribution were viewed using Tracer v1.7.2. Individual trees were visualized using FigTree v1.4.4 and the consensus tree was visualized using DensiTree v2.2.7

For manuscripts utilizing custom algorithms or software that are central to the research but not yet described in published literature, software must be made available to editors and reviewers. We strongly encourage code deposition in a community repository (e.g. GitHub). See the Nature Portfolio guidelines for submitting code & software for further information.

Data

Policy information about availability of data

All manuscripts must include a data availability statement. This statement should provide the following information, where applicable:

- Accession codes, unique identifiers, or web links for publicly available datasets
- A description of any restrictions on data availability
- For clinical datasets or third party data, please ensure that the statement adheres to our policy

All genomic reads generated during and/or analyzed during the current study are available under BioProject PRJNA741123 (http://www.ncbi.nlm.nih.gov/bioproject/741123). Other source data for the main figures can be found in Supplementary Data 1-5.

Field-specific reporting

Please select the one below that is the best fit for your research. If you are not sure, read the appropriate sections before making your selection.

☐ Life sciences ☐ Behavioural & social sciences ☒ Ecological, evolutionary & environmental sciences

For a reference copy of the document with all sections, see nature.com/documents/nr-reporting-summary-flat.pdf

Ecological, evolutionary & environmental sciences study design

All studies must disclose on these points even when the disclosure is negative.

Study description	To test models of ARO reservoir colonization and transmission in a hospital built-environment setting, we collected 1594 surface samples and 72 patient fecal samples at 24 time points from 6 ICU rooms in 2 buildings. Full metadata for 829 collected isolates has been included as Table S1. The first building was the SCT ICU that was located in a well-established hospital building, the "old ICU". The second was a newly constructed SCT ICU ("new ICU"); after construction was completed on the new ICU, the same staff and patients from the old ICU were all relocated to the new ICU. The old ICU rooms were sampled 3 times, with a week between samplings, during the final month of ICU occupancy (Figure 1B). New ICU rooms were sampled twice (two days apart) after the completion of construction while the rooms were unoccupied, then once every other week for the first 5 months of patient and staff occupancy (n=11 samplings), then once every month for the rest of the first year of occupancy (n=8 samplings) (Figure 1B). For both ICUs, we swabbed 10 high-touch ICU surfaces (with an additional 4 surfaces from attached bathrooms in new ICU rooms). We also obtained remnant fecal samples submitted for routine Clostridioides difficile testing as well as isolates recovered from standard-of-care blood cultures from patients in the ICU. We utilized selective microbiologic culture on surface and fecal samples to enrich for and culture AROs, including 1) organisms that form colonies on antibiotic media, which we later assess for resistance phenotypes by antibiotic susceptibility testing (AST), and 2) organisms that are inherently resistant to antibiotics, including Pseudomonas, Stenotrophomonas, and C. difficile. Blood culture isolates were recovered in the clinical laboratory as part of routine clinical methods (i.e. not selectively cultured for ARO) and were retrospectively obtained for during 46 different weeks of the study spanning 61 weeks total.
Research sample	We collected surface (see above), fecal, and blood isolates from at 24 time points from 6 ICU rooms in 2 buildings. The taxa identified from selective culture and genomic sequencing include 129 species denoted in Table S1. Fecal and blood isolates were retrospectively obtained from patients that resided in the wards sampled.
Sampling strategy	We sampled 6 SCT ICU (old ICU) rooms 3 times over the course of 1 month in the old building from December 2017 – January 2018. At each time point, nine surfaces were sampled using Eswab collections (Copan) pre-moistened with molecular water: the foam dispenser, the gown and glove storage area, the bedside rail, the nursing call button, the room floor, the light switch, the computer, the in-room sink handles, and the in-room sink drain. Three swabs were held together to simultaneously sample each surface. We also collected 2 samples of 15 mL in-room sink water directly from the faucet: 1 sample was collected immediately after turning the faucet on, and 1 sample was collected after allowing the water to run for 2 min.<br>We sampled 6 SCT ICU (new ICU) rooms and communal SCT ICU areas every other week for 5 months and then every month for 1 year in the new building for a total of 21 samplings (Figure 1B). Samples were collected twice during the first week of sample collections in the new ICU building: the first after construction terminal clean and the second after custodial terminal clean. Both time points collected were before patients and staff had entered the ICU. At each time point, the same nine patient room surfaces as described above were sampled plus an additional 3 surfaces: the sofa from the patient room, the bathroom toilet from adjoining bathroom, and the sink drain from the adjoining bathroom. We also collected 15 mL of in-room sink water and bathroom sink water. At each time point, we also sampled four communal surfaces: the housekeeping closet drain, the family area floor, the soiled utility room drain, and the vending machine. For each time point in both buildings, we obtained remnant de-identified fecal specimens that had been submitted to the clinical microbiology laboratory for C. difficile testing from patients in the same unit as surface swab collection.
Data collection	Eswab specimens from surfaces, water samples and fecal samples were cultured by members of the Burnham lab the same day of sampling Eswab specimens were vortexed and 90 µL of eluate was used for culture inoculation per plate/test condition. For fecal specimens, 90 µL of specimen was used for culture inoculation. For water samplings, 100µL of vortexed water sample was used for culturing. All samples were inoculated to each of the following culture medium: Sheep's blood agar (Hardy), VRE chromID (bioMerieux), Spectra MRSA (Remel), HardyCHROM ESBL (Hardy), MacConkey agar with cefotaxime (Hardy), Pseudo agar (Hardy), and Sabouraud dextrose + chloramphenicol (Hardy). Plates were incubated at 35oC in an air incubator and incubated up to 48h prior to discard if no growth (up to 7 days for sabouraud dextrose + chloramphenicol). Two colonies of each colony morphotype were subcultured and identified using matrix-assisted laser desorption/ionization time-of-flight mass spectrometry (MALD-TOF MS) with

the VITEK MS system. All isolates recovered were stored at -80oC in TSB with glycerol.
Antimicrobial susceptibility testing (AST) was performed using Kirby Bauer disk diffusion, interpreted according to CLSI standards. AST was performed on gram negative bacilli using ampicillin, cefazolin, cefotetan, ceftazidime, ceftriaxone, cefepime, meropenem, ciprofloxacin, levofloxacin, piperacillin-tazobactam, ceftolozane-tazobactam, ceftazidime-avibactam, ampicillin-sulbactam, trimethoprim-sulfamethoxazole, gentamicin, amikacin, fosfomycin, colistin, aztreonam, doxycycline, minocycline, doxycycline, and nitrofurantoin and antimicrobials were interpreted/reported as appropriate for the specific species. We also performed a carbapenamase inactivation assay on all Enterobacterales and Pseudomonas isolates that were resistant or intermediate to meropenem or imipenem.
Frozen isolates were passed to the Dantas lab for genomic sequencing. Total genomic DNA was extracted from cultured isolates using the Bacteremia kit (Qiagen, Gernmantown, MD, USA) DNA was quantified using the PicoGreen dsDNA assay (Thermo Fisher Scientific, Waltham, MA, USA). A total of 5 ng/µL was used as input for Illumina sequencing libraries with the Nextera kit (Illumina, San Diego, CA, USA). The libraries were pooled and sequenced on a NextSeq HighOutput platform (Illumina) to obtain 2x150bp reads.

Timing and spatial scale: See sampling strategy.

Data exclusions: No data was excluded in this study.

Reproducibility: The authors involved have a history of successful environmental sampling. We measured the same rooms multiple times longitudinally and used 3 swabs at every sampling location, and have stored replicate swabs for future study.

Randomization: Randomization is not applicable to this study.

Blinding: Blinding is not applicable to this study.

Did the study involve field work? ☐ Yes ☒ No

Reporting for specific materials, systems and methods

We require information from authors about some types of materials, experimental systems and methods used in many studies. Here, indicate whether each material, system or method listed is relevant to your study. If you are not sure if a list item applies to your research, read the appropriate section before selecting a response.

Materials & experimental systems

n/a	Involved in the study	
☒	☐	Antibodies
☒	☐	Eukaryotic cell lines
☒	☐	Palaeontology and archaeology
☒	☐	Animals and other organisms
☐	☒	Human research participants
☒	☐	Clinical data
☒	☐	Dual use research of concern

Methods

n/a	Involved in the study	
☒	☐	ChIP-seq
☒	☐	Flow cytometry
☒	☐	MRI-based neuroimaging

Human research participants

Policy information about studies involving human research participants

Population characteristics: Patient blood cultures were retrospectively obtained from routine clinical care. Patient blood isolates were selected if they resided within the sampled wards during the time the environmental sampling strategy took place.

Recruitment: Patient blood isolates were selected if they resided within the sampled wards during the time the environmental sampling strategy took place. All samples fitting the described criteria were utilized.

Ethics oversight: The collection and analyses of patient blood culture isolates in this study was approved by the Institutional Review Board of Washington University School of Medicine (IRB 201901053) and by the Siteman Cancer Center's Protocol Review and Monitoring Committee. We received IRB approval and Siteman Cancer approval for clinical isolates from patients.

Note that full information on the approval of the study protocol must also be provided in the manuscript.
