# Supplementary material for: Antibiotic-resistant organisms establish reservoirs in new hospital built environments and are related to patient blood infection isolates
Source: Commun Med (Lond). 2022 Jun 1;2:62. doi: 10.1038/s43856-022-00124-5 (PMC9160058; doi:10.1038/s43856-022-00124-5)
Supplement: Supplementary file 8 — Supplementary Information [file 43856_2022_124_MOESM8_ESM.pdf]

Supplementary Information

Antibiotic-resistant organisms establish reservoirs in new hospital built environments and are related to patient blood infection isolates.

Kimberley V. Sukhum[1,2,^], Erin P. Newcomer[1,2,3^], Candice Cass[4], Meghan A. Wallace[2], Caitlin Johnson[2], Jeremy Fine[2], Steven Sax[4], Margaret H. Barlet[4], Carey-Ann D. Burnham[2,4,5,6*], Gautam Dantas[1,2,3,5*], and Jennie H. Kwon[4*]

[1] The Edison Family Center for Genome Sciences and Systems Biology, Washington University School of Medicine in St Louis, St Louis, MO, USA
[2] Department of Pathology and Immunology, Washington University School of Medicine in St Louis, St Louis, MO, USA
[3] Department of Biomedical Engineering, Washington University in St Louis, St Louis, MO, USA
[4] Department of Medicine, Washington University School of Medicine in St Louis, St Louis, MO, USA
[5] Department of Molecular Microbiology, Washington University School of Medicine in St Louis, St Louis, MO, USA
[6] Department of Pediatrics, Washington University School of Medicine in St Louis, St Louis, MO, USA

[^]These authors contributed equally to this work.
[*]Correspondence: CDB, cburnham@wustl.edu. GD, dantas@wustl.edu. JK, j.kwon@wustl.edu.

This PDF contains:
Supplementary Figures 1–5.

Supplementary Figures

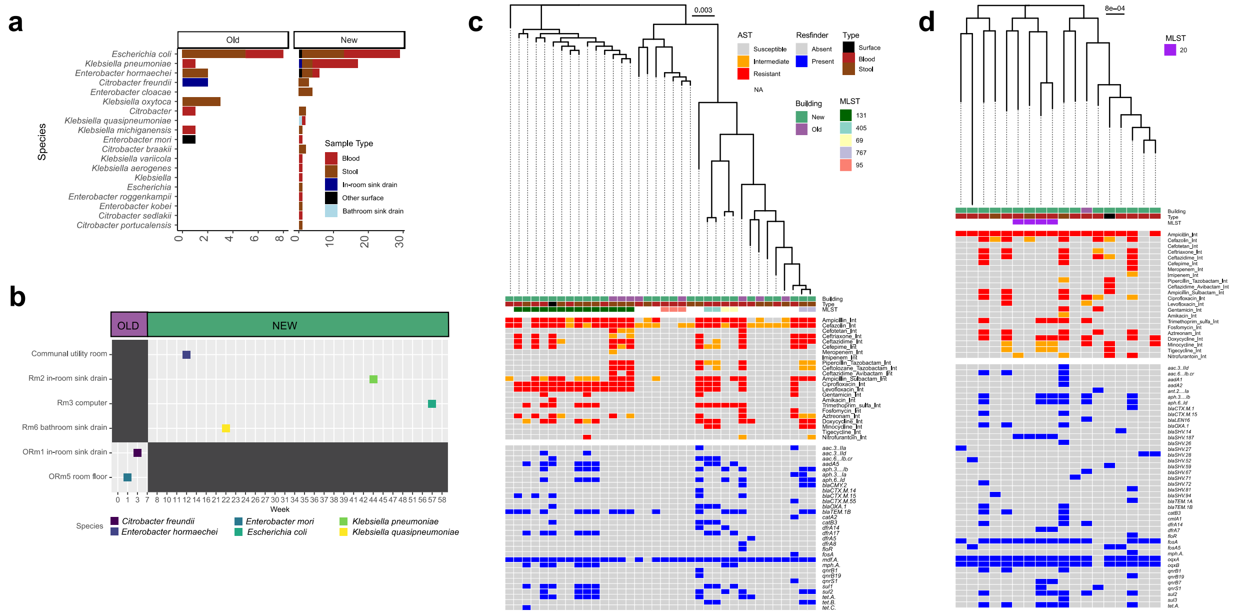

Supplementary Figure 1: Identity, timing, and resistance of Enterobacterales isolates. **a** Identity of all collected Enterobacterales genomes by >95% average nucleotide identify (ANI) to reference genome, colored by sample collection type. Other indicates all other surface/water genomes apart from in-room and bathroom sink drain. All genomes were identified to genus by MASH. **b** Time point mapping of all antibiotic-resistant (AR) Enterobacterales isolates cultured from surface samples by sample collection location. Dark grey boxes indicate no surface collections. Purple point indicates 2 morphotypes of *Citrobacter freundii*. **c** Phenotypic and genotypic antibiotic resistance of collected *E. coli* isolates. Phylogenetic tree is from a core genome alignment. Branches with low bootstrap values are shown and tree has resolution of 0.00055. Phenotypic resistance determined by antibiotic susceptibility testing (AST). Genotypic resistance determined by Resfinder[1]. **d** Phenotypic and genotypic antibiotic resistance of collected *K. pneumoniae* isolates. Phylogenetic tree is from a core genome alignment and has resolution of 0.00055. Phenotypic resistance determined by AST. Genotypic resistance determined by Resfinder[1].

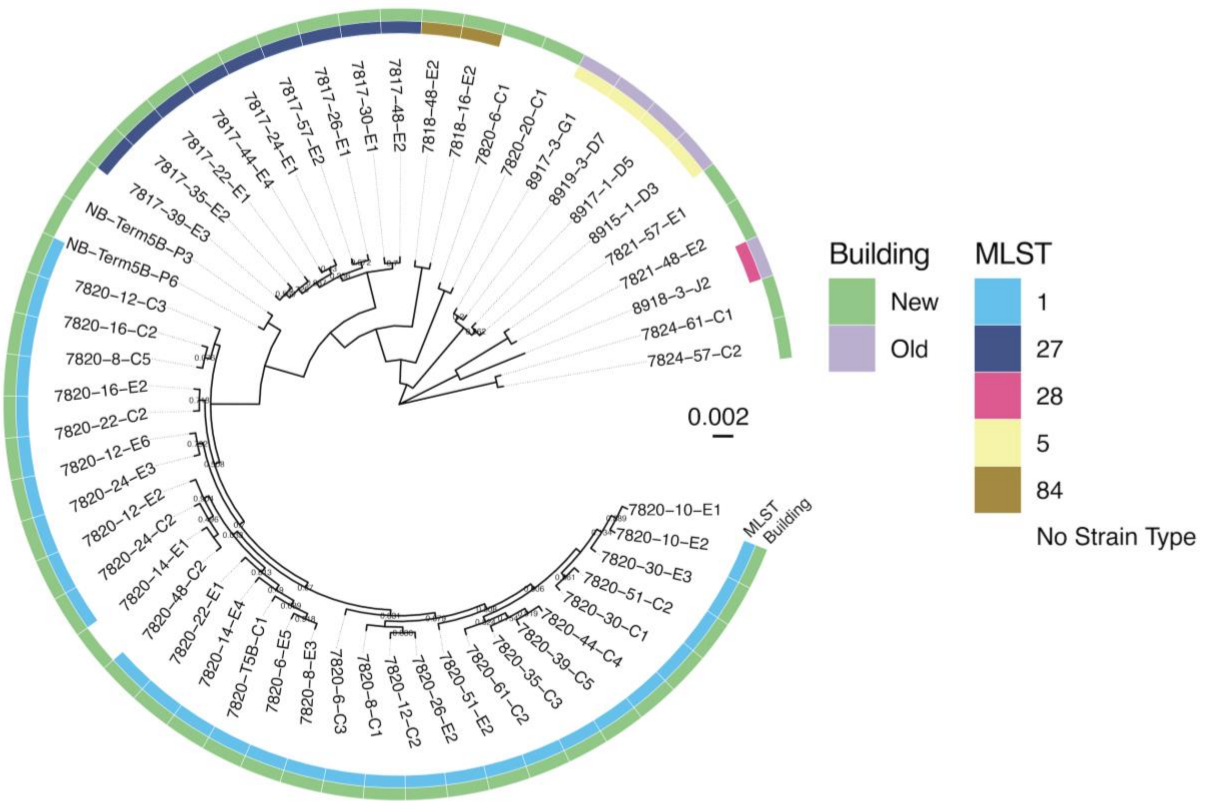

Supplementary Figure 2: Phylogram built from core genome alignments of *S. maltophilia* genomes.

Branches with low bootstrap values are shown, minimum resolution of 0.00055.

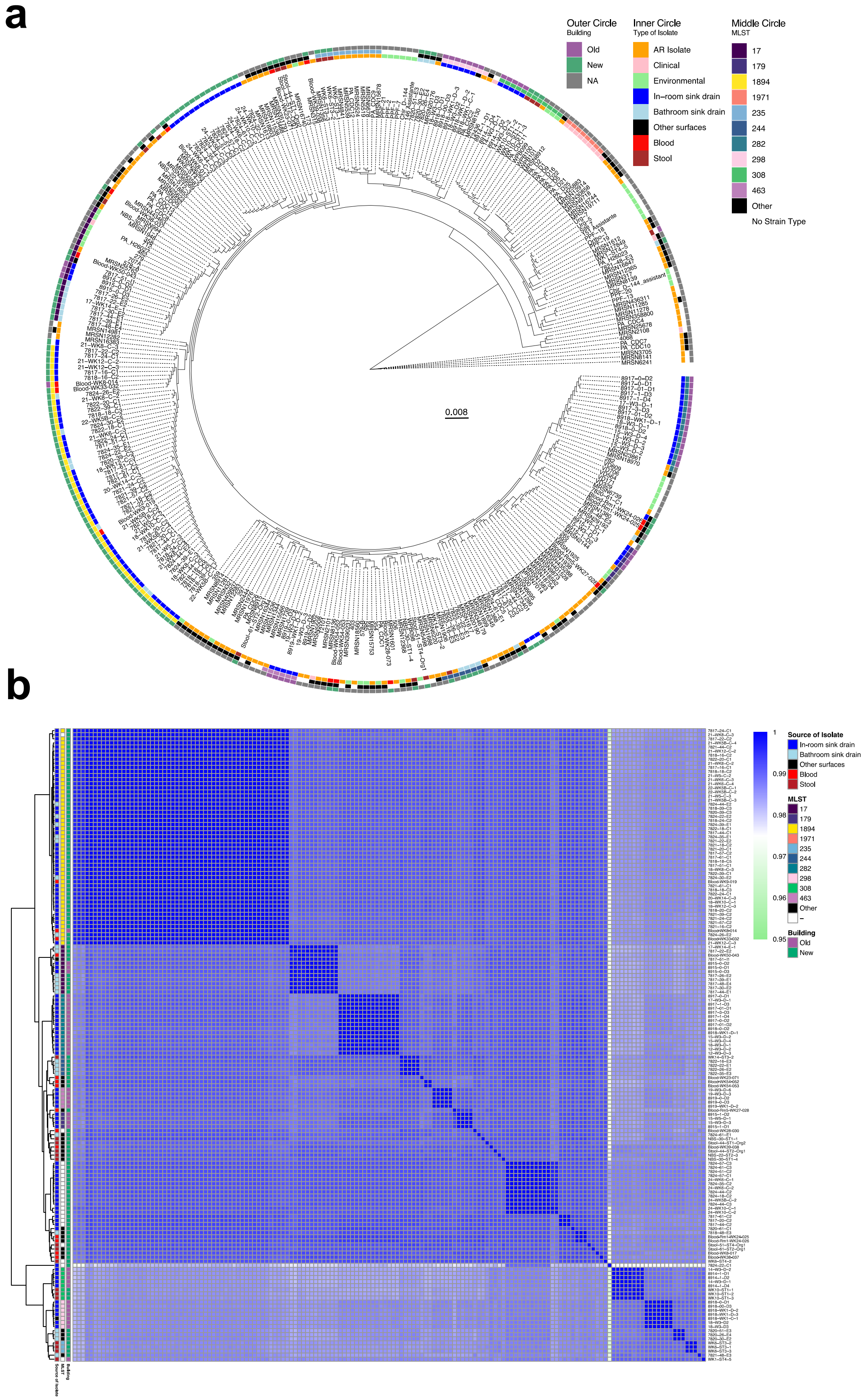
a
Outer Circle
Building
Old
New
NA
Inner Circle
Type of Isolate
AR Isolate
Clinical
Environmental
In–room sink drain
Bathroom sink drain
Other surfaces
Blood
Stool
Middle Circle
MLST
17
179
1894
1971
235
244
282
298
308
463
Other
No Strain Type
0.008
b
Source of Isolate
In-room sink drain
Bathroom sink drain
Other surfaces
Blood
Stool
MLST
17
179
1894
1971
235
244
282
298
308
463
Other
–
Building
Old
New
1
0.99
0.98
0.97
0.96
0.95
Source of Isolate
MLST
Building

Supplementary Figure 3: Average nucleotide identity (ANI) and phylogenetics of *P. aeruginosa* genomes.

a Phylogram built from core genome alignments of *P. aeruginosa* genomes. Branches with low bootstrap values are shown, minimum resolution of 0.00055. **b** Average nucleotide identify (ANI) heatmap of all *P. aeruginosa* genomes.

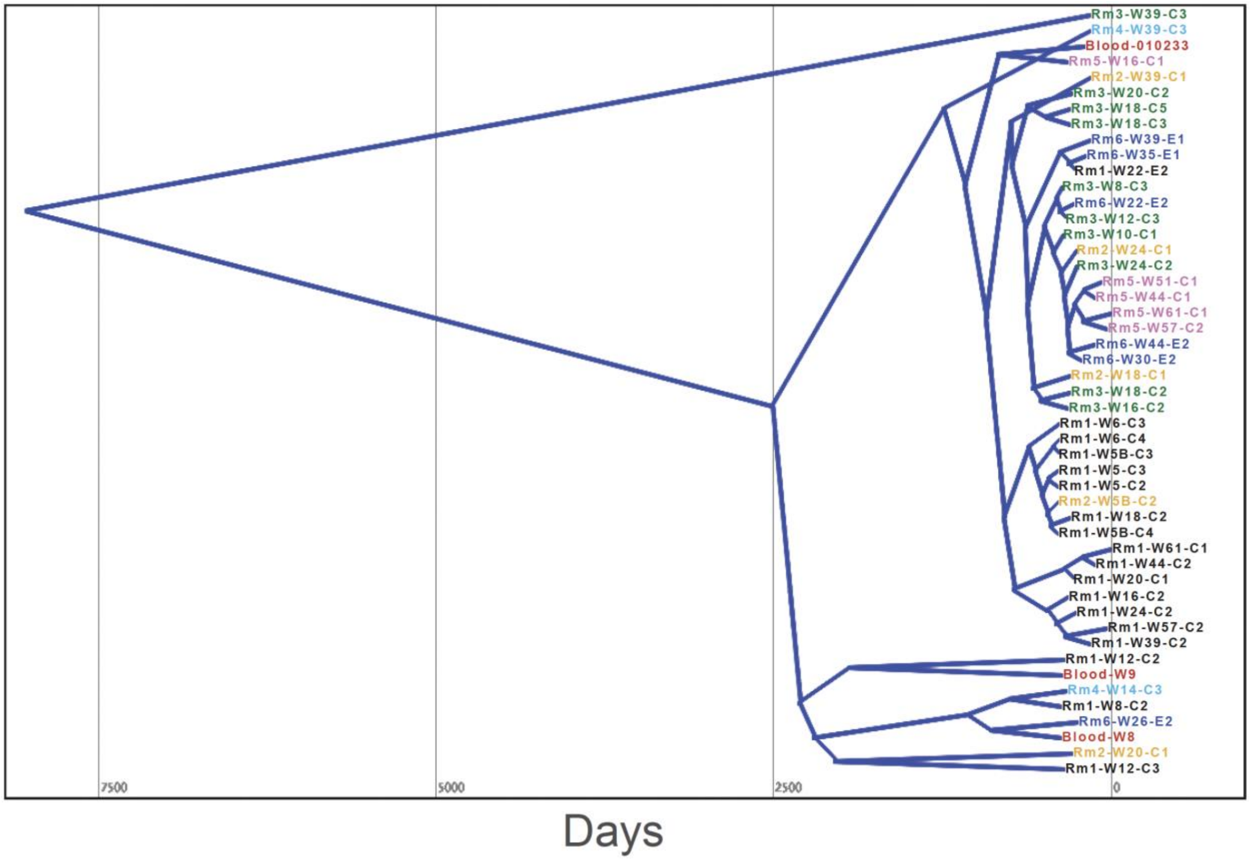

Supplementary Figure 4: Time-measured phylogenetic analysis consensus tree of Group 1 *P. aeruginosa* isolates.

Tree of n=49 isolates depicted using DensiTree v2.2.7[2]. 4 Group 1 isolates were removed from this tree because they represented a significant portion of the accessory genome and were unlikely part of the same lineage. The most distant isolate (Rm3-W39-C3) was excluded from further analysis.

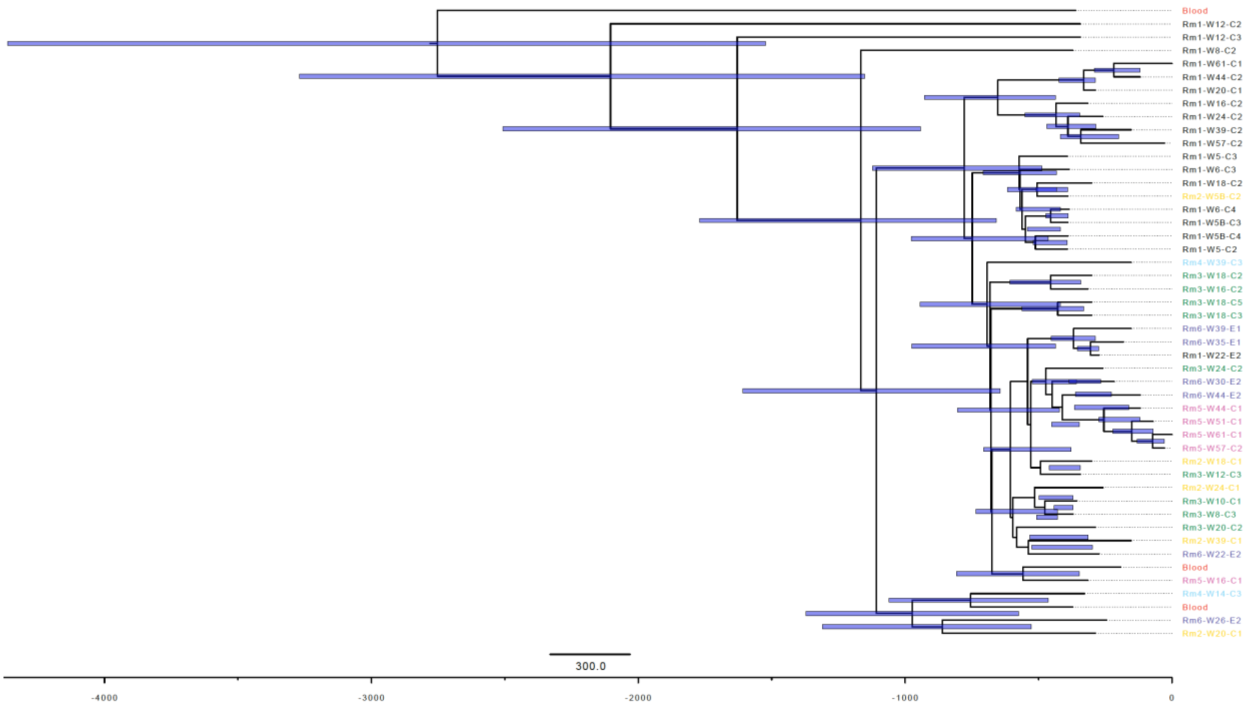

Supplementary Figure 5: Time-measured phylogenetic analysis consensus tree of Group 1 *P. aeruginosa* isolates.

Tree of n=48 isolates depicted using FigTree v1.4.4[3]. 4 Group 1 isolates were removed from this tree because they represented a significant portion of the accessory genome and were unlikely part of the same lineage. One isolate was removed because it was highly divergent from all other remaining isolates. Error bars represent the 95% highest posterior density interval (HPD) interval for node height. Uncertainty is higher for earlier time since most recently ancestors (TMRCAs) because they are outside of the sampling range.

Supplementary References

1 Zankari, E. *et al.* Identification of acquired antimicrobial resistance genes. *J Antimicrob Chemother* **67**, 2640-2644, doi:10.1093/jac/dks261 (2012).

2 Bouckaert, R. & Heled, J. DensiTree 2: Seeing Trees Through the Forest. *bioRxiv* (2014).

3 Rambaut, A., Suchard, M., Nenarokov, S. & Klotzl, F. FigTree. (2018).
